# Body Mass Index-Adjusted Weight Loss Grading System and Cancer-Related Fatigue in Survivors 1 Year After Esophageal Cancer Surgery

**DOI:** 10.1245/s10434-022-11633-x

**Published:** 2022-04-01

**Authors:** Zhao Cheng, Poorna Anandavadivelan, Magnus Nilsson, Asif Johar, Pernilla Lagergren

**Affiliations:** 1 Surgical Care Science, Department of Molecular Medicine and Surgery, Karolinska Institutet, Karolinska University Hospital, Stockholm, Sweden; 2 Division of Surgery, Department of Clinical Science, Intervention and Technology (CLINTEC), Karolinska Institutet, Stockholm, Sweden; 3 Department of Upper Abdominal Diseases, Karolinska University Hospital, Stockholm, Sweden; 4 Department of Surgery and Cancer, Imperial College London, London, UK

## Abstract

**Background:**

The association between pre- and postoperative weight loss and cancer-related fatigue after esophageal cancer surgery is unclear. This nationwide, prospective, longitudinal cohort study aimed to assess the influence of weight loss on cancer-related fatigue among esophageal cancer survivors.

**Methods:**

Patients who underwent esophagectomy for cancer between 2013 and 2019 in Sweden were enrolled in this study. Exposure was measured by the body mass index-adjusted weight loss grading system (WLGS). Cancer-related fatigue was assessed using the fatigue scale of the European Organization for Research and Treatment of Cancer Quality of Life Questionnaire Core 30 (EORTC QLQ-C30) and the EORTC QLQ-Fatigue 12 (QLQ-FA12) questionnaire measuring overall fatigue and physical, emotional, and cognitive fatigue. Growth mixture models were used to identify unobserved trajectories of cancer-related fatigue. Multivariable linear and logistic regression models were fitted to assess the associations between WLGS and cancer-related fatigue, adjusting for potential confounders.

**Results:**

Three trajectories were identified—low, moderate, and severe persistent fatigue. Cancer-related fatigue remained stable in each trajectory between 1 and 3 years after esophagectomy. Among the 356 enrolled patients, 4.5–22.6% were categorized into the severe persistent fatigue trajectory in terms of QLQ-C30 (19.9%), FA12 overall (10.5%), physical (22.6%), emotional (15.9%), and cognitive fatigue (4.5%). No association between pre- or postoperative WLGS and cancer-related fatigue was found between 1 and 3 years after esophageal cancer surgery.

**Conclusions:**

Weight loss did not seem to influence cancer-related fatigue after esophageal cancer surgery.

**Supplementary Information:**

The online version contains supplementary material available at 10.1245/s10434-022-11633-x.

Weight loss is a major concern for esophageal cancer patients. According to a previous Swedish cohort study, approximately one-fifth of esophageal cancer patients lost more than 10% of their average weight before curatively intended esophagectomy and one-third of patients lost over 15% of their average weight within 6 months after surgery.^1,2^

The lack of a clinically relevant definition of weight change has been a barrier for studies regarding weight among cancer patients. Arbitrarily defined cut-offs, regardless of the initial body habitus, inhibit the interpretation and comparison of study results. To address this, the body mass index (BMI)-adjusted weight loss grading system (WLGS) was proposed in 2015 as a validated classification by combining weight change and BMI,^3^ and has been proven to have prognostic validity regarding survival and quality of life in cancer patients.^4,5^

Cancer-related fatigue is the subjective feeling of physical, emotional, and cognitive exhaustion related to both cancer and cancer treatment that cannot be alleviated by rest or sleep.^6,7^ It is one of the most reported quality-of-life symptoms of esophageal cancer patients.^8,9^ The mechanism and risk factors for cancer-related fatigue are largely unknown and reliable data from prospective, longitudinal studies are scarce. Recognized risk factors include baseline fatigue level and postoperative complications.^10–12^ Unintentional weight loss in esophageal cancer patients has been found to be associated with a reduced response to treatment, poor prognosis, and decreased quality of life;^13^ however, little is known about the consequences of weight loss on cancer-related fatigue. Therefore, this study aimed to explore the association between pre- or postoperative weight loss and cancer-related fatigue using the WLGS in a longitudinal design. Such knowledge could help identify patients with a high risk of postoperative cancer-related fatigue in need of support and provide suggestions and evidence for planning future studies.

## Material and Methods

### Study Design

This study was built on an ongoing Swedish nationwide, prospective cohort entitled ‘Oesophageal Surgery on Cancer patients—Adaptation and Recovery (OSCAR) study’, detailed information about which has been previously published.^8,12^ Briefly, OSCAR includes all esophageal cancer survivors who underwent esophagectomy between 1 January 2013 and 30 June 2020 in Sweden. Patients are followed up regularly from 1 to 12 years after surgery. For the purpose of this study, patients who underwent esophagectomy between January 2013 and December 2019 were enrolled and all available data up to and including the 3-year follow-up were used. The project was approved by the Regional Ethical Review Board in Stockholm and informed consent forms were obtained from all participants.

### Data Source and Data Collection

Patients were identified via pathology centers in Sweden and those who survived 1 year after esophagectomy were included. Cancer-related fatigue was measured at 1, 1.5, 2, 2.5, and 3 years postoperatively. At 1 year, patients were visited by a research nurse who collected the data via computer-based questionnaires. For the other follow-ups, patients responded to paper questionnaires. To diminish the influence of cancer recurrence, fatigue measurements from patients who died within 2 months of the last interview were excluded. Clinical data at the time of surgery, including tumor histology, pathological tumor stage, treatment, and postoperative complications were collected by review of medical records (histopathology reports, operation charts, and discharge notes), and data on comorbidities were extracted from the Swedish Patient Registry.^14^ Weight at the time of operation was collected from medical records, while the average weight as an adult, weight at 6 months postoperatively, and height of the patient as an adult were reported by patients at the postoperative 1-year interview using the Patient-Generated Subjective Global Assessment (PG-SGA) questionnaire.^15^ Data linkages of participants were enabled by the Swedish unique personal identity number, a 10-digit number assigned to each Swedish resident.^16^

### Exposure

The study exposure was BMI-adjusted WLGS, as summarized in Table 1.^3^ BMI was calculated as [current weight (kg)/height (m^2^)], whereas weight loss (%) was calculated as [(current weight (kg) − previous weight (kg))/previous weight (kg)] * 100.

**Table 1:** Body mass index-adjusted weight loss grading system (0–4)

Weight loss (%)	Body mass index (kg/m^2^)				
	≥ 28	25–28	22–25	20–22	< 20
< 2.5	0	0	1	1	3
2.5–6	1	2	2	2	3
6–11	2	3	3	3	4
11–15	3	3	3	4	4
≥ 15	3	4	4	4	4

Preoperative WLGS was categorized by BMI at operation and weight loss between average weight as an adult and at the time of operation; postoperative WLGS was categorized by BMI at 6 months after surgery and weight loss between weight at operation and 6 months after surgery; and cumulative WLGS was categorized by BMI at 6 months after surgery and weight loss between average weight as an adult and weight at 6 months after surgery.

### Outcome

The primary outcome was cancer-related fatigue score at 1 year after esophagectomy (continuous variable), and the secondary outcome was cancer-related fatigue trajectory membership (categorical variable).

Cancer-related fatigue was measured by two validated questionnaires: fatigue subscale of the European Organization for Research and Treatment of Cancer Quality of Life Core Questionnaire (EORTC QLQ-C30) and the EORTC QLQ-Fatigue (EORTC QLQ-FA12).^17,18^ The EORTC QLQ-C30 is a 30-item questionnaire that evaluates quality of life in cancer patients and includes a three-item subscale measuring cancer-related fatigue. EORTC QLQ-FA12 is a multidimensional instrument measuring overall, physical, emotional, and cognitive aspects of cancer-related fatigue along with EORTC QLQ-C30. Questionnaire-measured fatigue scores were transformed into 0–100 scales. Missing data were handled according to the EORTC scoring manual. A higher score represents more cancer-related fatigue.

### Statistical Analysis

Growth mixture models were used to identify unobserved trajectories of cancer-related fatigue among esophageal cancer patients, an approach that aims to identify homogeneous subgroups within a heterogeneous population.^10,19–21^ Cancer-related fatigue trajectories were estimated and compared between models with 1–4 latent classes and with different model complexities (zero-order, linear, quadratic, and cubic splines). Model selection was based on Akaike Information Criterion (AIC), Bayesian Information Criterion (BIC), sample size-adjusted BIC, trajectory sample size, and model interpretability. Patients were assigned to each trajectory according to the group membership probability calculated from the models. All available data were integrated into growth mixture models using full-information maximum likelihood estimation based on the assumption that data are missing at random (MAR).

Linear regression models were fitted to calculate the mean score and mean score difference of cancer-related fatigue at 1 year after esophagectomy with 95% confidence intervals (CI) for patients with different WLGS, adjusting for confounders. Clinical relevance was defined as a difference in mean scores of at least 5 on the transformed scale.^22,23^ Logistic regression models were fitted to calculate odds ratios (OR) with 95% CIs to assess the association between WLGS and cancer-related fatigue trajectories, adjusting for confounding factors. Additionally, sensitivity analyses were conducted among patients with dumping syndrome and among patients who were involved in the Enhanced Recovery After Surgery (ERAS) program.^24^

The confounding factors used in the linear and logistic regression models were age at surgery (continuous variable), sex (male or female), pathological tumor stage (0–I, II or III–IV), neoadjuvant therapy (no or yes), Charlson Comorbidity Index (0, 1, or ≥ 2),^25^ and tumor histology (adenocarcinoma or squamous cell carcinoma). When analyzing the association between postoperative or cumulative WLGS and cancer-related fatigue, in addition to the previous confounders, Clavien–Dindo classifications (0–I, II–IIIa, or IIIb–IV)^26^ were also included as a confounder.

To further elucidate the association between weight change and cancer-related fatigue, a post hoc analysis was conducted; weight loss and BMI were included in the regression models separately.

An experienced biostatistician (AJ) was responsible for the statistical analyses. SAS 9.4 software (SAS Institute Inc., Cary, NC, USA) was used for all analyses.

## Results

### Patients

In Sweden, 921 patients underwent esophagectomy for esophageal cancer between January 2013 and December 2019. Among these patients, 221 (24.0%) died within 1 year and 131 (14.2%) were not reachable, resulting in 569 patients being eligible for inclusion in this study, of whom 356 (62.6%) completed the 1-year fatigue assessment. Patients did not participate due to the following reasons: they were too sick (57, 10.0%), were unwilling to participate (128, 22.5%), or there was a lack of information on clinical, sociodemographic, or fatigue factors (28, 4.9%). At 1.5, 2, 2.5, and 3 years, 328, 299, 246, and 213 of the 356 patients were alive and passed the time for follow-up, and 82.6% (271/328), 76.6% (229/299), 67.5% (166/246), 68.1% (145/213) responded to the follow-up questionnaires, respectively. Table 2 describes the patient characteristics according to the postoperative WLGS. The mean age at operation was 67.2 years. Most patients were male (90.4%), had adenocarcinoma (85.1%), and underwent neoadjuvant therapy (78.9%).

**Table 2:** Characteristics of the 356 patients who underwent esophagectomy for esophageal cancer categorized by postoperative WLGS

	Total	Postoperative WLGS				
		0	1	2	3	4
Total	356 (100.0)	20 (100.0)	28 (100.0)	41 (100.0)	141 (100.0)	114 (100.0)
*Age at operation*						
Mean (SD)	67.2 (8.4)	67.6 (9.3)	66.4 (8.7)	66.5 (11.0)	67.3 (7.7)	67.5 (8.2)
*Sex*						
Female	34 (9.6)	4 (20.0)	6 (21.4)	1 (2.4)	10 (7.1)	12 (10.5)
Male	322 (90.4)	16 (80.0)	22 (78.6)	40 (97.6)	131 (92.9)	102 (89.5)
*Pathological tumor stage*						
0–I	121 (34.0)	5 (25.0)	9 (32.1)	9 (22.0)	51 (36.2)	42 (36.8)
II	110 (30.9)	7 (35.0)	11 (39.3)	13 (31.7)	39 (27.7)	34 (29.8)
III–IV	125 (35.1)	8 (40.0)	8 (28.6)	19 (46.3)	51 (36.2)	38 (33.3)
*Tumor histological type*						
Adenocarcinoma	303 (85.1)	16 (80.0)	19 (67.9)	35 (85.4)	124 (87.9)	98 (86.0)
Squamous cell carcinoma	53 (14.9)	4 (20.0)	9 (32.1)	6 (14.6)	17 (12.1)	16 (14.0)
*Neoadjuvant therapy*						
No	75 (21.1)	6 (30.0)	6 (21.4)	6 (14.6)	30 (21.3)	24 (21.1)
Yes	281 (78.9)	14 (70.0)	22 (78.6)	35 (85.4)	111 (78.7)	90 (78.9)
*Charlson Comorbidity Index*						
0	149 (41.9)	5 (25.0)	11 (39.3)	20 (48.8)	63 (44.7)	46 (40.4)
1	119 (33.4)	9 (45.0)	7 (25.0)	12 (29.3)	49 (34.8)	39 (34.2)
≥ 2	88 (24.7)	6 (30.0)	10 (35.7)	9 (22.0)	29 (20.6)	29 (25.4)
*Clavien–Dindo classification*						
None–I	134 (37.6)	7 (35.0)	6 (21.4)	17 (41.5)	61 (43.3)	38 (33.3)
II–IIIa	131 (36.8)	6 (30.0)	14 (50.0)	15 (36.6)	52 (36.9)	41 (36.0)
IIIb–IV	91 (25.6)	7 (35.0)	8 (28.6)	9 (22.0)	28 (19.9)	35 (30.7)

*WLGS* body mass index-adjusted weight loss grading system, *SD* standard deviation

### Cancer-Related Fatigue Trajectories

**Fig. 1:**
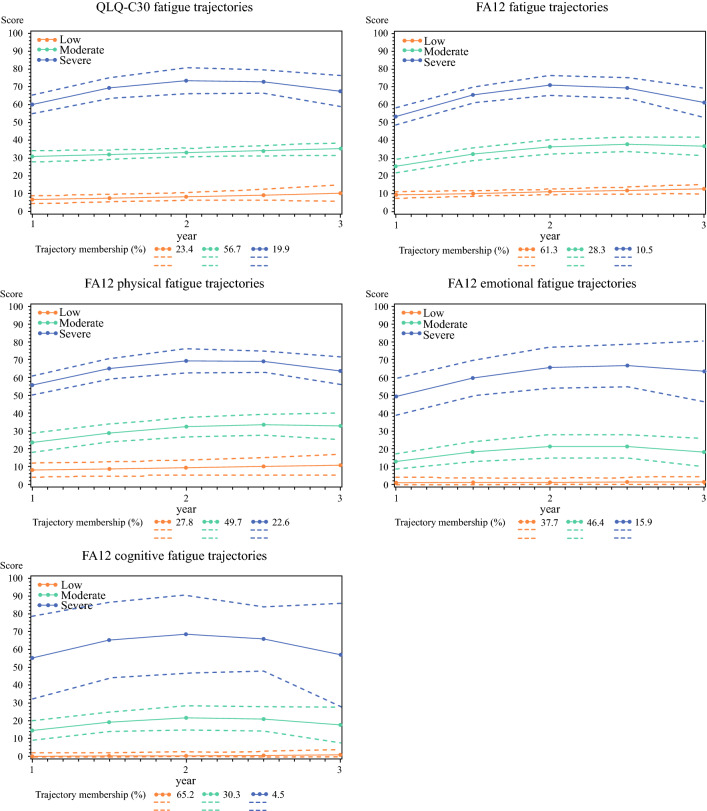
Cancer-related fatigue trajectories between 1 and 3 years after esophagectomy for esophageal cancer. *QLQ-C30* Quality of Life Questionnaire Core 30, *FA12* 12-item fatigue questionnaire

Based on the model comparison and practical interpretation, the three-class models were selected for all fatigue scores and patients were grouped into three fatigue trajectories—low, moderate, and severe persistent fatigue. Figure 1 presented the mean scores, with 95% CIs, for the identified fatigue trajectories. Membership of the severe persistent fatigue trajectory was regarded as the outcome in the logistic regression. Some 4.5–22.6% of patients were categorized into the severe persistent fatigue trajectory in terms of QLQ-C30 (19.9%), FA12 overall (10.5%), physical (22.6%), emotional (15.9%), and cognitive fatigue (4.5%). Fit statistics for model comparison are presented in the electronic supplementary material (ESM; Table S1 and Fig. S1).

The trajectories of cancer-related fatigue measured with different questionnaires were similar. In general, the three trajectories were stable through the study period, especially for patients in the low persistent fatigue trajectory group, while the fatigue score of patients from the moderate and severe persistent trajectory groups increased slightly over a 1- to 2-year period and then reverted back to the initial level 3 years after esophagectomy.

### Weight Loss Grading System (WLGS) and Cancer-Related Fatigue

Before esophageal cancer surgery, about one-third of patients had the lowest WLGS of 0 (34.5%), and after esophagectomy, the majority of patients (70.5%) had the worst WLGS of 3–4 [Table 4].

Cancer-related fatigue scores at 1 year after esophagectomy were similar across patients with different preoperative WLGSs. No changing pattern was found for the mean difference estimates comparing higher grade with grade 0. Furthermore, neither clinical relevance nor statistical significance was found for fatigue score change in patients with different postoperative and cumulative WLGSs (Table 3).

**Table 3:** Adjusted cancer-related fatigue scores and mean differences with 95% confidence intervals among patients with different WLGS at 1 year after esophagectomy

	QLQ-C30 fatigue	QLQ-FA12 fatigue	QLQ-FA12 physical fatigue	QLQ-FA12 emotional fatigue	QLQ-FA12 cognitive fatigue
*Preoperative WLGS*					
0	35.9 (29.9–41.8)	21.5 (17.1–25.9)	30.3 (24.4–36.1)	17.7 (12.2–23.2)	10.1 (6.1–14.1)
1 versus 0 MD	− 5.3 (− 15.2 to 4.7)	1.1 (− 6.2 to 8.5)	− 0.9 (− 10.6 to 8.9)	3.8 (− 5.3 to 12.9)	3.6 (− 3.0 to 10.3)
2 versus 0 MD	2.3 (− 6.5 to 11.1)	1.1 (− 5.5 to 7.7)	1.5 (− 7.2 to 10.1)	1.2 (− 6.8 to 9.3)	− 2.2 (− 8.1 to 3.7)
3 versus 0 MD	− 3.1 (− 11.7 to 5.5)	− 2.1 (− 8.5 to 4.3)	− 3.4 (− 11.8 to 5.0)	− 1.1 (− 8.9 to 6.8)	− 1.3 (− 7.0 to 4.5)
4 versus 0 MD	2.8 (− 8.5 to 14.1)	− 0.7 (− 9.1 to 7.7)	− 1.6 (− 12.7 to 9.5)	− 0.1 (− 10.4 to 10.3)	2.8 (− 4.8 to 10.3)
*Postoperative WLGS*^a^					
0	28.8 (18.3–39.4)	24.3 (16.4–32.2)	31.0 (20.6–41.5)	26.0 (16.4–35.7)	15.8 (8.8–22.9)
1 versus 0 MD	7.9 (− 7.8 to 23.6)	− 4.1 (− 15.9 to 7.7)	− 4.5 (− 20.1 to 11.1)	− 7.6 (− 21.9 to 6.8)	− 4.8 (− 15.3 to 5.8)
2 versus 0 MD	1.9 (− 12.9 to 16.7)	− 4.3 (− 15.4 to 6.8)	− 3.4 (− 18.1 to 11.3)	− 9.0 (− 22.5 to 4.5)	− 4.4 (− 14.3 to 5.5)
3 versus 0 MD	7.4 (− 5.6 to 20.4)	− 2.6 (− 12.3 to 7.2)	− 0.1 (− 13.0 to 12.8)	− 8.6 (− 20.5 to 3.2)	− 5.3 (− 14.0 to 3.4)
4 versus 0 MD	5.0 (− 8.1 to 18.1)	− 2.1 (− 11.9 to 7.8)	0.6 (− 12.4 to 13.7)	− 7.2 (− 19.2 to 4.8)	− 5.3 (− 14.1 to 3.5)
*Cumulative WLGS*^a^					
0	36.7 (25.9–47.5)	26.2 (18.2–34.3)	34.5 (23.9–45.1)	25.3 (15.5–35.2)	15.0 (7.7–22.3)
1 versus 0 MD	− 0.3 (− 17.4 to 16.8)	− 3.2 (− 16.0 to 9.5)	− 5.5 (− 22.3 to 11.3)	− 2.0 (− 17.6 to 13.6)	0.6 (− 11.0 to 12.1)
2 versus 0 MD	− 0.4 (− 16.0 to 15.2)	− 3.8 (− 15.5 to 7.8)	− 2.8 (− 18.2 to 12.5)	− 5.3 (− 19.6 to 8.9)	− 4.3 (− 14.9 to 6.2)
3 versus 0 MD	− 4.8 (− 18.0 to 8.4)	− 7.6 (− 17.4 to 2.3)	− 8.3 (− 21.3 to 4.6)	− 10.4 (− 22.4 to 1.7)	− 6.0 (− 14.9 to 2.9)
4 versus 0 MD	− 2.1 (− 14.9 to 10.7)	− 5.4 (− 14.9 to 4.2)	− 5.2 (− 17.7 to 7.4)	− 7.1 (− 18.7 to 4.6)	− 4.9 (− 13.5 to 3.7)

Adjusted for age at surgery, sex, pathological tumor stage, neoadjuvant therapy, Charlson Comorbidity Index, and tumor histology

*WLGS* body mass index-adjusted weight loss grading system, *MD* mean differences, *QLQ-C30* Quality of Life Questionnaire Core 30, *QLQ-FA12* Quality of Life Questionnaire Fatigue 12

^a^Further adjusted for Clavien–Dindo classification

After grouping patients into different cancer-related fatigue trajectories, patients with a higher WLGS showed no increased risk of having the severe persistent fatigue trajectory, which applied to preoperative, postoperative, and cumulative WLGSs (Table 4).

**Table 4:** WLGS and ORs with 95% CIs for severe cancer-related fatigue trajectory membership after esophagectomy

	*N* (%)^a^	QLQ-C30 fatigue	QLQ-FA12 fatigue	QLQ-FA12 physical fatigue	QLQ-FA12 emotional fatigue	QLQ-FA12 cognitive fatigue
		[OR (95% CI)]	[OR (95% CI)]	[OR (95% CI)]	[OR (95% CI)]	[OR (95% CI)]
*Preoperative WLGS*						
0	125 (34.5)	1.0 (Reference)	1.0 (Reference)	1.0 (Reference)	1.0 (Reference)	1.0 (Reference)
1	44 (12.2)	0.93 (0.37–2.34)	1.78 (0.60–5.29)	1.09 (0.48–2.44)	1.83 (0.72–4.67)	1.41 (0.29–6.95)
2	61 (16.9)	0.75 (0.31–1.78)	1.51 (0.53–4.32)	1.06 (0.51–2.21)	1.69 (0.72–4.01)	0.42 (0.04–4.16)
3	67 (18.5)	1.03 (0.47–2.29)	0.70 (0.21–2.42)	0.66 (0.30–1.44)	0.93 (0.36–2.42)	0.38 (0.04–3.78)
4	33 (9.1)	0.93 (0.33–2.61)	1.79 (0.52–6.21)	0.55 (0.19–1.60)	1.27 (0.42–3.81)	2.08 (0.38–11.51)
*Postoperative WLGS*^b^						
0	20 (5.5)	1.0 (Reference)	1.0 (Reference)	1.0 (Reference)	1.0 (Reference)	1.0 (Reference)
1	28 (7.7)	1.87 (0.31–11.27)	1.08 (0.14–8.26)	1.03 (0.26–4.08)	0.46 (0.10–2.13)	0.58 (0.06–5.83)
2	41 (11.3)	1.48 (0.26–8.62)	0.84 (0.11–6.30)	0.55 (0.13–2.21)	0.56 (0.13–2.37)	0.32 (0.02–4.73)
3	141 (39.0)	2.76 (0.58–13.16)	1.52 (0.28–8.24)	1.09 (0.35–3.38)	0.67 (0.20–2.22)	0.64 (0.09–4.30)
4	114 (31.5)	1.90 (0.39–9.24)	1.81 (0.33–9.76)	1.08 (0.35–3.37)	0.73 (0.22–2.44)	0.45 (0.06–3.11)
*Total cumulative WLGS*^b^						
0	21 (5.8)	1.0 (Reference)	1.0 (Reference)	1.0 (Reference)	1.0 (Reference)	1.0 (Reference)
1	19 (5.2)	2.03 (0.38–10.78)	2.23 (0.29–17.41)	0.43 (0.10–1.91)	1.23 (0.25–6.13)	4.78 (0.32–71.36)
2	28 (7.7)	2.76 (0.61–12.45)	1.73 (0.26–11.56)	0.73 (0.21–2.52)	0.76 (0.17–3.31)	2.55 (0.19–33.35)
3	108 (29.8)	0.90 (0.22–3.73)	0.62 (0.10–3.83)	**0.33 (0.11**–**0.98)**	0.37 (0.10–1.39)	0.49 (0.03–7.99)
4	152 (42.0)	1.23 (0.32–4.72)	1.22 (0.23–6.48)	0.48 (0.18–1.33)	0.58 (0.17–1.95)	0.96 (0.08–11.07)

Bold values indicate statistical significance (*p* < 0.05)

Adjusted for age at surgery, sex, pathological tumor stage, neoadjuvant therapy, Charlson Comorbidity Index, and tumor histology

*WLGS* body mass index-adjusted weight loss grading system, *ORs* odds ratios, *CIs* confidence intervals, *QLQ-C30* Quality of Life Questionnaire Core 30, *QLQ-FA12* Quality of Life Questionnaire Fatigue 12

^a^Percentages do not add up to 100% due to missing data

^b^Further adjusted for Clavien–Dindo classification

The lack of association remained in the sensitivity analysis among patients with dumping syndrome and those who were involved in the ERAS program (data not shown).

In the post hoc analysis, after adjusting for weight loss, both pre- and postoperative BMI <20 was associated with an increased level of first-year cancer-related fatigue, as well as the risk of being in the severe persistent trajectory (ESM Tables S2 and S3).

## Discussion

This study did not support the hypothesis of an increased risk of cancer-related fatigue for patients with higher WLGS after esophagectomy for esophageal cancer.

Methodological strengths of this study include the population-based cohort design with prospective longitudinal follow-up and robust assessment of exposures and outcomes. The combination measurements using two validated questionnaires and trajectory identification of cancer-related fatigue, including both overall and dimensional scores, provide a comprehensive understanding of the symptom. However, several issues should be kept in mind in view of interpretation of the results. Although the grading system considered initial habitus (BMI), body composition was not incorporated in WLGS and the impact from weight change due to sarcopenia or muscle loss could not be detected. Another limitation arises from the inevitable residual confounding in this observational study setting, such as information on change of lifestyle and cancer recurrence. However, the exclusion of measurements from patients who died within 2 months of the last questionnaire measurement should have alleviated the concerns about tumor recurrence to some extent. Selection bias could have also influenced the results since the characteristics of patients who were not reachable or declined to participate are unknown. Furthermore, patients who did not participate due to unwillingness and serious illness might have severe weight loss and fatigue, hence the potential associations would be diluted. Moreover, few patients had severe preoperative or minor postoperative weight loss in this cohort; thus, the lack of association must be interpreted cautiously. Lastly, statistical power is another issue in this study. Despite the longitudinal nationwide study design, the low incidence and high mortality of esophageal cancer^27^ put restrictions on the big sample size and some mild effects are difficult to detect. In addition, some patients had not passed the later follow-ups (e.g. patients who underwent surgery at the end of 2019 did not reach the 3-year follow-up at the time of data analysis).

To our knowledge, this is the first study identifying cancer-related fatigue trajectories among esophageal cancer patients. The number of trajectories was comparable with studies conducted among patients with other tumor types. The shape of each trajectory was rather flat, especially for patients among the low persistent fatigue trajectories 1–3 years after esophagectomy. In Germany, one longitudinal study identified three to four cancer-related fatigue trajectories among 4215 Hodgkin’s lymphoma survivors with different tumor stages, and each fatigue trajectory also remained stable between 1 and 5 years after treatment.^10^ Another French cohort study revealed three to five trajectories in different fatigue dimensions among 459 female breast cancer patients, and the fatigue levels persisted within each trajectory despite transient fluctuations during 2 years after surgery.^28^ The reasons for the stable fatigue level over time in the severe fatigue group could be explained not only by patients dying but also other reasons that are more difficult to account for, e.g. response shift with better adaptation or coping strategy. Furthermore, the relative stabilization of the cancer-related fatigue trajectory indicated the potential of using the initial measurement to identify patients with a higher probability of having a long-term fatigue burden.

Mechanisms for cancer-related fatigue are multifactorial and the most known biological explanation states that cancer-related fatigue comes from inflammatory processes. In line with this, many of the identified risk factors are associated with elevated inflammation activities, such as comorbidities and nutritional issues.^29^ BMI and weight loss are common and easy-to-use nutritional indicators for esophageal cancer patients. Pre-^30–32^ and postoperative^33,34^ weight loss might be associated with prognosis after esophagectomy independent of BMI. Higher WLGS has been found to increase the risk of poor quality of life, including cancer-related fatigue, in patients with incurable cancers^5^. Thus, it was intuitive to assume that underweight esophageal cancer patients with severe weight loss had a higher level of cancer-related fatigue, which was surprisingly not found in the current study.

One possible explanation could be that the inflammation associated with weight loss might only explain a limited share of cancer-related fatigue in the cohort. The reasons for weight loss in esophageal cancer patients are complex, including cancer cachexia, eating difficulty, and postoperative changes in eating habits and gastrointestinal hormone feedback balance.^27,35^ Among these, the most theoretically feasible reason related to inflammation and cancer-related fatigue is cancer cachexia, which is not a dominating symptom in this patient group.^13^ While other nutrition-related reasons, such as dysphagia and dumping syndrome, might not impact fatigue directly, which was also seen in the sensitivity analysis, the risk of fatigue did not increase among patients with dumping syndrome and with recovery support. The second explanation is about the potential role of muscle loss in maintaining energy. WLGS does not incorporate body composition and sarcopenic patients may be more likely to have cancer-related fatigue. Preoperative sarcopenia has been shown to be associated with major postoperative complications in esophageal cancer patients,^36^ which is also supportive evidence of this explanation since complications have been identified as risk factors for cancer-related fatigue.^11,12,37^

In the post hoc analysis, a potential influence from low BMI on increased cancer-related fatigue was found after adjusting for weight loss. However, in studies of breast cancer, BMI was not found as a predictor for fatigue trajectory after surgery (no adjustment for weight change).^28,38^ Differences in tumor types and fatigue measurements might explain the varying findings, particularly since the logic and concerns for weight loss and BMI are not the same for different cancer types.

## Conclusion

This nationwide, longitudinal study provided no evidence of considering weight loss as a risk factor for cancer-related fatigue among esophageal cancer patients. Larger studies are warranted for further investigation. This is important information for understanding cancer-related fatigue and the conclusion is valuable for future study planning about cancer-related fatigue.

## Supplementary Information

Below is the link to the electronic supplementary material.

Supplementary file1 (DOCX 575 kb)
