# Supplementary material for: Body Mass Index-Adjusted Weight Loss Grading System and Cancer-Related Fatigue in Survivors 1 Year After Esophageal Cancer Surgery
Source: Ann Surg Oncol. 2022 Apr 1;29(7):4502–10. doi: 10.1245/s10434-022-11633-x (PMC9174120; doi:10.1245/s10434-022-11633-x)
Supplement: Supplementary file 1 — Supplementary file1 (DOCX 575 kb) [file 10434_2022_11633_MOESM1_ESM.docx]

**Supplementary Information:**

| **Table S1. Model comparison fit statistics for cancer-related fatigue trajectories** | | | | |
| --- | --- | --- | --- | --- |
| **Number of classes** | **Trajectory shapes** | **BIC1** | **BIC2** | **AIC** |
| Model comparison for QLQ-C30 fatigue | | |  |  |
| 1 | 2 | -4779.85 | -4782.21 | -4772.12 |
| 2 | 2 2 | -4638.66 | -4643.38 | -4623.19 |
| 2 | 1 2 | -4635.74 | -4639.88 | -4622.21 |
| 3 | 2 2 2 | -4545.03 | -4552.12 | -4521.83 |
| 3 | 1 1 2 | -4539.62 | -4545.53 | -4520.29 |
| 4 | 2 2 2 2 | -4539.8 | -4549.24 | -4508.86 |
| 4 | 1 1 2 2 | -4534.17 | -4542.44 | -4507.1 |
| 5 | 2 2 2 2 2 | -4531.8 | -4543.61 | -4493.13 |
| Model comparison for QLQ-FA12 fatigue | |  |  |  |
| 1 | 2 | -4700.28 | -4702.64 | -4692.55 |
| 2 | 2 2 | -4440.33 | -4445.03 | -4424.86 |
| 2 | 1 2 | -4439.63 | -4443.75 | -4426.1 |
| 3 | 1 1 2 | -4349.99 | -4355.87 | -4330.66 |
| 3 | 2 2 2 | -4349.8 | -4356.86 | -4326.6 |
| 3 | 1 2 2 | -4348.65 | -4355.12 | -4327.38 |
| 4 | 2 2 2 2 | -4312.16 | -4321.57 | -4281.22 |
| 4 | 1 1 2 2 | -4310.14 | -4318.37 | -4283.07 |
| 5 | 2 2 2 2 2 | -4305.27 | -4317.03 | -4266.6 |
| Model comparison for QLQ-FA12 physical fatigue | | |  |  |
| 1 | 2 | -4789.21 | -4791.56 | -4781.47 |
| 2 | 2 2 | -4608.94 | -4613.64 | -4593.47 |
| 2 | 1 2 | -4607.79 | -4611.9 | -4594.25 |
| 3 | 2 2 2 | -4549.84 | -4556.89 | -4526.64 |
| 3 | 1 2 2 | -4546.95 | -4553.41 | -4525.68 |
| 4 | 2 2 2 2 | -4511.73 | -4521.13 | -4480.8 |
| 4 | 1 1 2 2 | -4508.76 | -4516.99 | -4481.7 |
| 5 | 2 2 2 2 2 | -4512.31 | -4524.06 | -4473.64 |
| Model comparison for QLQ-FA12 emotional fatigue | | |  |  |
| 1 | 2 | -3368.14 | -3370.49 | -3360.41 |
| 2 | 2 2 | -3217.39 | -3222.09 | -3201.93 |
| 2 | 1 2 | -3216.1 | -3220.22 | -3202.57 |
| 3 | 2 2 2 | -3153.98 | -3161.03 | -3130.78 |
| 3 | 1 2 2 | -3151.23 | -3157.7 | -3129.96 |
| 4 | 2 2 2 2 | -3139.58 | -3148.98 | -3108.65 |
| 4 | 1 2 2 2 | -3136.98 | -3145.8 | -3107.98 |
| 5 | 2 2 2 2 2 | -3147.36 | -3159.12 | -3108.7 |
| Model comparison for QLQ-FA12 cognitive fatigue | | |  |  |
| 1 | 2 | -1960.25 | -1962.6 | -1952.51 |
| 2 | 2 2 | -1804.42 | -1809.13 | -1788.95 |
| 2 | 1 2 | -1802.42 | -1806.54 | -1788.89 |
| 3 | 2 2 2 | -1799.08 | -1806.14 | -1775.89 |
| 3 | 1 2 2 | -1785.58 | -1792.05 | -1764.31 |
| 4 | 2 2 2 2 | -1790.04 | -1799.45 | -1759.1 |
| 4 | 1 1 2 2 | -1785.54 | -1793.78 | -1758.47 |
| Trajectory shapes: 0 = zero-order; 1 = linear; 2 = quadratic; 3 = cubic  BIC1: Bayesian information criterion (sample size adjusted BIC)  BIC2: Bayesian information criterion  AIC: Akaike Information Criterion  Green-marked models were presented in Figure 1. Yellow-marked models were presented in Figure S1. | | | | |

Although the four-class model (Figure S1) had lower AIC and BIC, the three-class model was selected due to the following reasons:

- Average class membership probabilities were higher for the three-class model.
- More than 10% participants in each trajectory (except cognitive fatigue).
- To avoid the overlap of the trajectory confidence intervals in the four-class model.


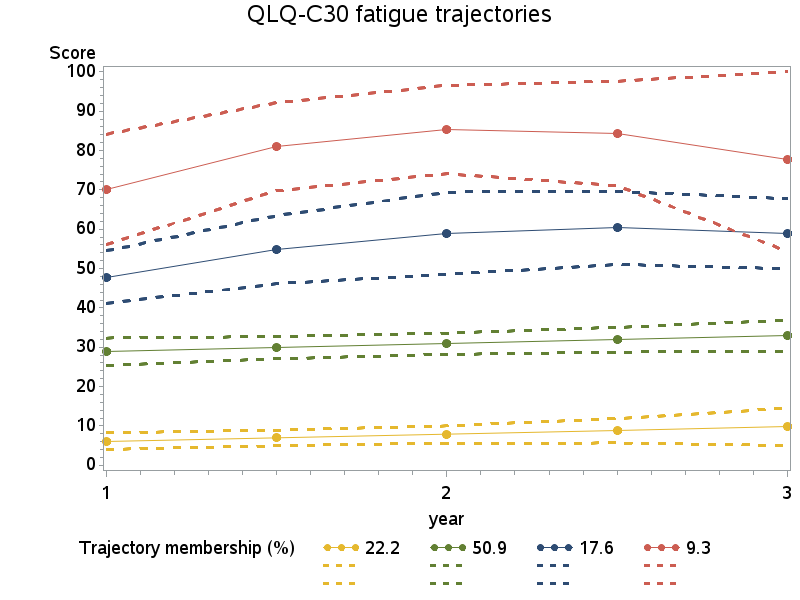

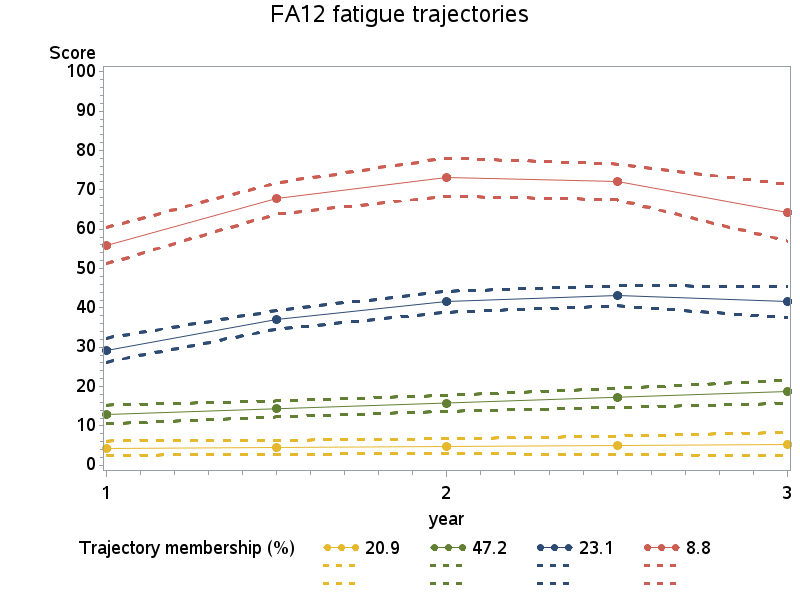

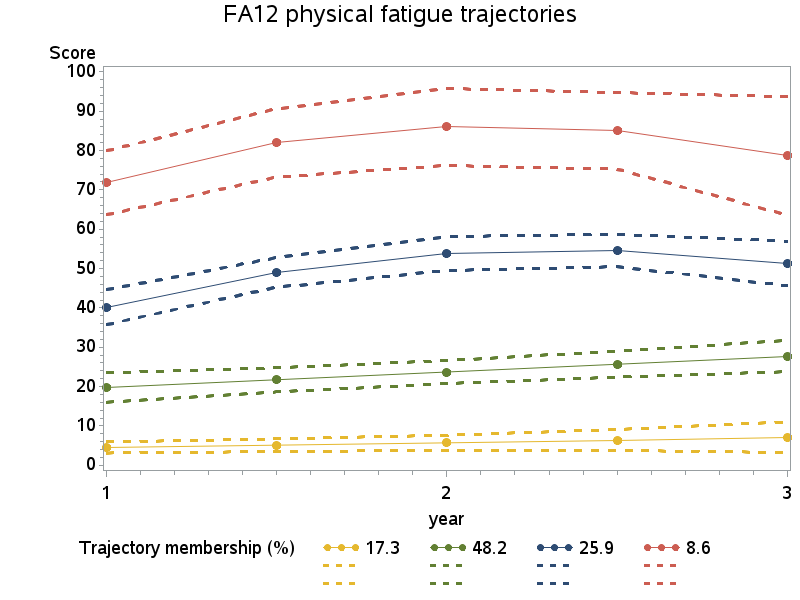

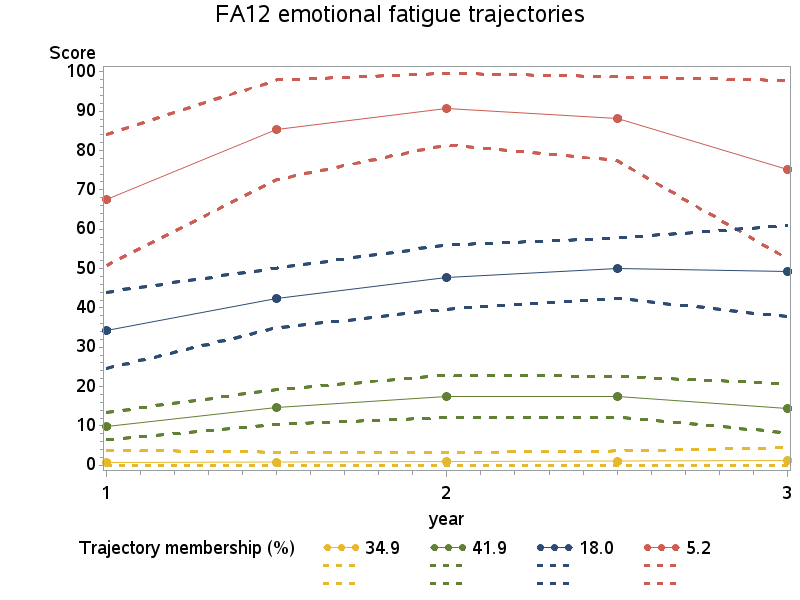

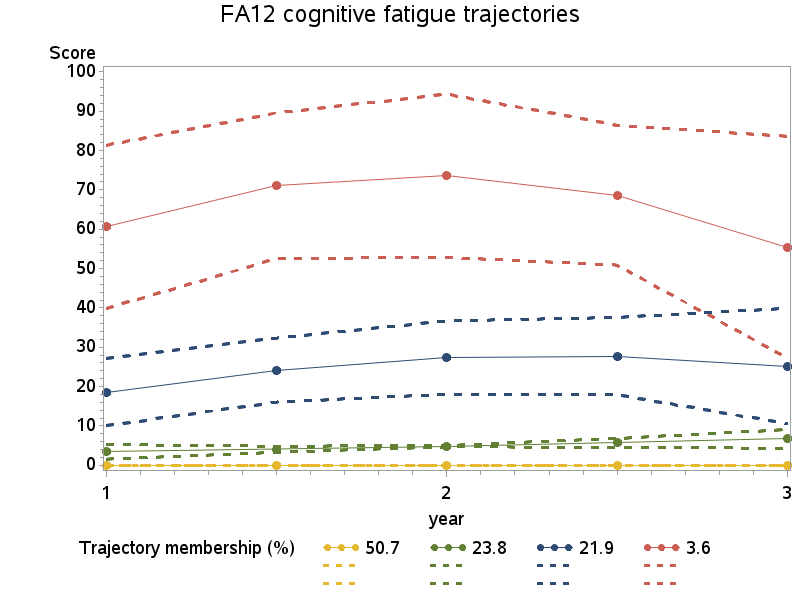


**Figure S1. Cancer-related fatigue trajectories between 1 and 3 years after esophagectomy for esophageal cancer**

| **Table S2. Adjusted^1^ cancer-related fatigue scores and mean differences (MDs) with 95% confidence intervals (CIs) among patients with different weight loss and** **body mass index (BMI) at one year after esophagectomy** | | | | | |  |
| --- | --- | --- | --- | --- | --- | --- |
|  | **QLQ-C30 fatigue** | **QLQ-FA12 fatigue** | **QLQ-FA12 physical fatigue** | **QLQ-FA12 emotional fatigue** | **QLQ-FA12 cognitive fatigue** | |
| **Preoperative weight loss (%)^2^** | |  |  |  |  | |
| < 2.5 | 35.2 (29.5-40.8) | 22.0 (17.8-26.2) | 30.5 (25.0-36.0) | 18.7 (13.6-23.8) | 11.1 (7.3-14.8) | |
| 2.5-6 VS < 2.5 MD | 1.8 (-6.8-10.4) | 2.6 (-3.8-8.9) | 2.5 (-5.8-10.9) | 3.2 (-4.6-11.0) | 0.7 (-5.1-6.4) | |
| 6-11 VS < 2.5 MD | -0.2 (-8.9-8.5) | -1.7 (-8.1-4.7) | -2.9 (-11.3-5.6) | -1.0 (-8.9-6.9) | -2.3 (-8.2-3.5) | |
| 11-15 VS < 2.5 MD | 0.0 (-13.4-13.5) | -4.1 (-14.0-5.8) | -3.7 (-16.8-9.4) | -4.6 (-16.8-7.7) | -3.0 (-12.1-6.0) | |
| ≥ 15 VS < 2.5 MD | -4.9 (-16.8-6.9) | -5.4 (-14.1-3.4) | -8.0 (-19.6-3.5) | -4.0 (-14.7-6.8) | 0.9 (-7.0-8.9) | |
| **Preoperative BMI^3^** |  |  |  |  |  | |
| ≥ 28 | 31.1 (24.9-37.3) | 18.4 (13.8-23.0) | 25.9 (19.8-31.9) | 15.8 (10.1-21.5) | 8.0 (3.8-12.2) | |
| 25-28 VS ≥ 28 MD | 4.3 (-3.5-12.1) | 2.6 (-3.1-8.4) | 2.7 (-4.9-10.2) | 1.5 (-5.7-8.6) | 4.1 (-1.1-9.3) | |
| 22-25 VS ≥ 28 MD | 3.4 (-5.3-12.1) | 3.5 (-3.0-9.9) | 4.6 (-3.8-13.1) | 3.0 (-5.0-11.0) | 3.1 (-2.7-9.0) | |
| 20-22 VS ≥ 28 MD | 8.1 (-4.4-20.7) | 3.1 (-6.2-12.3) | 4.3 (-7.9-16.5) | 1.9 (-9.6-13.4) | 0.1 (-8.3-8.6) | |
| < 20 VS ≥ 28 MD | **14.5 (-1.5-30.5)** | **12.3 (0.5-24.2)** | **15.0 (-0.5-30.5)** | 12.5 (-2.2-27.1) | **9.5 (-1.2-20.3)** | |
| **Postoperative weight loss (%)^4^** | |  |  |  |  | |
| < 2.5 | 36.2 (28.8-43.6) | 24.0 (18.4-29.5) | 30.6 (23.2-37.9) | 24.7 (18.0-31.5) | 14.1 (9.2-19.1) | |
| 2.5-6 VS < 2.5 MD | -2.7 (-14.6-9.2) | -2.3 (-11.3-6.6) | -0.3 (-12.2-11.5) | -7.4 (-18.3-3.4) | -2.4 (-10.3-5.6) | |
| 6-11 VS < 2.5 MD | -2.9 (-13.1-7.3) | -2.1 (-9.8-5.6) | -0.1 (-10.3-10.1) | -6.0 (-15.3-3.4) | -2.3 (-9.1-4.6) | |
| 11-15 VS < 2.5 MD | 3.1 (-7.5-13.7) | -0.8 (-8.8-7.2) | 2.5 (-8.1-13.1) | -6.3 (-16.0-3.4) | -3.5 (-10.7-3.6) | |
| ≥ 15 VS < 2.5 MD | -5.6 (-16.0-4.9) | -5.0 (-12.9-2.8) | -2.6 (-12.9-7.8) | **-9.7 (-19.3--0.2)** | -5.9 (-12.9-1.1) | |
| **Postoperative BMI^5^** |  |  |  |  |  | |
| ≥ 28 | 31.1 (23.2-39.0) | 18.7 (12.8-24.6) | 26.3 (18.5-34.1) | 16.1 (8.9-23.3) | 9.6 (4.3-14.9) | |
| 25-28 VS ≥ 28 MD | 3.6 (-7.3-14.4) | 4.8 (-3.2-12.9) | 5.9 (-4.8-16.6) | 5.2 (-4.6-15.0) | 2.3 (-4.9-9.6) | |
| 22-25 VS ≥ 28 MD | -0.0 (-9.9-9.8) | 0.2 (-7.1-7.6) | 0.4 (-9.3-10.1) | -0.7 (-9.6-8.3) | 0.2 (-6.4-6.8) | |
| 20-22 VS ≥ 28 MD | 8.4 (-2.4-19.2) | 4.7 (-3.3-12.8) | 6.9 (-3.7-17.6) | 4.3 (-5.5-14.1) | 1.5 (-5.7-8.8) | |
| < 20 VS ≥ 28 MD | 8.9 (-3.3-21.2) | **8.7 (-0.4-17.9)** | **11.7 (-0.4-23.8)** | 8.5 (-2.6-19.6) | 4.1 (-4.1-12.3) | |
| **Cumulative weight loss (%)^4^** | |  |  |  |  | |
| < 2.5 | 36.4 (28.0-44.9) | 25.5 (19.2-31.8) | 33.2 (24.9-41.6) | 25.8 (18.1-33.5) | 14.3 (8.6-20.0) | |
| 2.5-6 VS < 2.5 MD | -1.6 (-15.1-11.9) | -5.1 (-15.2-5.0) | -4.9 (-18.2-8.4) | -8.3 (-20.6-4.1) | -1.3 (-10.4-7.8) | |
| 6-11 VS < 2.5 MD | 0.1 (-11.8-12.0) | -3.1 (-12.0-5.7) | -3.2 (-14.9-8.5) | -5.7 (-16.5-5.2) | -5.4 (-13.4-2.7) | |
| 11-15 VS < 2.5 MD | -4.9 (-16.5-6.7) | -6.9 (-15.5-1.8) | -6.5 (-17.9-4.9) | **-10.4 (-21.0-0.1)** | -5.6 (-13.4-2.2) | |
| ≥ 15 VS < 2.5 MD | -3.1 (-13.6-7.4) | -6.0 (-13.8-1.8) | -5.5 (-15.8-4.8) | **-9.6 (-19.2--0.1)** | -4.1 (-11.2-3.0) | |
| The category of weight loss and BMI are the same as in the BMI adjusted weight loss grading system.  1 Adjusted for age at surgery, sex, pathological tumor stage, neoadjuvant therapy, Charlson comorbidity index, and tumor histology.  2 Further adjust for preoperative BMI.  3 Further adjust for preoperative weight loss.  4 Further adjust for postoperative BMI and Clavien-Dindo classification.  5 Further adjust for postoperative weight loss and Clavien-Dindo classification. | | | | | |  |

| **Table S3. Adjusted^1^ cancer-related fatigue scores and mean differences (MDs) with 95% confidence intervals (CIs) among patients with different weight loss and** **body mass index (BMI) at one year after esophagectomy** | | | | | |
| --- | --- | --- | --- | --- | --- |
|  | **QLQ-C30 fatigue** | **QLQ-FA12 fatigue** | **QLQ-FA12 physical fatigue** | **QLQ-FA12 emotional fatigue** | **QLQ-FA12 cognitive fatigue** |
| **Preoperative weight loss (%)^2^** | |  |  |  |  |
| < 2.5 | 1.0 (Reference) | 1.0 (Reference) | 1.0 (Reference) | 1.0 (Reference) | 1.0 (Reference) |
| 2.5-6 | 1.28 (0.75-2.17) | 1.57 (0.61-4.04) | 1.71 (0.86-3.38) | 1.89 (0.86-4.19) | 0.29 (0.03-2.75) |
| 6-11 | 1.01 (0.59-1.72) | 0.70 (0.23-2.16) | 0.72 (0.33-1.57) | 1.12 (0.47-2.69) | 0.32 (0.03-2.91) |
| 11-15 | 1.00 (0.44-2.30) | 0.45 (0.05-3.89) | 0.54 (0.14-2.05) | 0.26 (0.03-2.15) | 0.82 (0.07-9.36) |
| ≥ 15 | 0.66 (0.32-1.37) | 0.75 (0.17-3.22) | 0.32 (0.09-1.21) | 0.73 (0.21-2.58) | 1.16 (0.18-7.57) |
| **Preoperative BMI^3^** | |  |  |  |  |
| ≥ 28 | 1.0 (Reference) | 1.0 (Reference) | 1.0 (Reference) | 1.0 (Reference) | 1.0 (Reference) |
| 25-28 | 1.51 (0.92-2.45) | 1.06 (0.37-3.01) | 1.20 (0.61-2.36) | 0.85 (0.36-2.01) | 7.84 (1.12-54.96) |
| 22-25 | 1.44 (0.83-2.49) | 2.60 (0.87-7.82) | 1.16 (0.53-2.54) | 2.27 (0.94-5.48) | 6.19 (0.71-53.76) |
| 20-22 | **2.15 (0.98-4.74)** | 1.47 (0.25-8.82) | 0.87 (0.25-3.00) | 1.83 (0.49-6.77) | -**^6^** |
| < 20 | **2.73 (1.00-7.45)** | **9.85 (1.95-49.77)** | 3.06 (0.79-11.77) | **4.39 (1.05-18.37)** | **32.22 (2.35-442.36)** |
| **Postoperative weight loss (%)^4^** | |  |  |  |  |
| < 2.5 | 1.0 (Reference) | 1.0 (Reference) | 1.0 (Reference) | 1.0 (Reference) | 1.0 (Reference) |
| 2.5-6 | 0.90 (0.42-1.94) | 0.66 (0.15-2.92) | 1.06 (0.36-3.06) | 0.31 (0.09-1.11) | 1.11 (0.16-7.49) |
| 6-11 | 0.82 (0.42-1.58) | 1.03 (0.32-3.33) | 1.25 (0.51-3.06) | 0.82 (0.31-2.15) | 0.53 (0.09-3.07) |
| 11-15 | 1.36 (0.69-2.70) | 0.61 (0.16-2.33) | 0.78 (0.29-2.07) | 0.64 (0.23-1.83) | 0.25 (0.02-2.87) |
| ≥ 15 | 0.65 (0.33-1.28) | 0.82 (0.24-2.75) | 1.05 (0.42-2.62) | 0.44 (0.16-1.26) | 0.36 (0.06-2.16) |
| **Postoperative BMI^5^** | |  |  |  |  |
| ≥ 28 | 1.0 (Reference) | 1.0 (Reference) | 1.0 (Reference) | 1.0 (Reference) | 1.0 (Reference) |
| 25-28 | 1.31 (0.65-2.65) | 2.18 (0.48-9.99) | 1.37 (0.53-3.56) | 1.38 (0.46-4.13) | 3.04 (0.38-24.47) |
| 22-25 | 0.91 (0.48-1.71) | 2.41 (0.57-10.15) | 0.91 (0.37-2.24) | 0.76 (0.26-2.22) | 1.39 (0.15-12.67) |
| 20-22 | **2.11 (1.05-4.26)** | 1.12 (0.22-5.80) | 1.46 (0.56-3.79) | 1.18 (0.38-3.59) | 0.83 (0.05-12.72) |
| < 20 | **2.05 (0.93-4.53)** | **8.05 (1.71-37.84)** | 1.58 (0.54-4.61) | 3.01 (0.92-9.84) | 9.69 (0.90-104.00) |
| **Cumulative weight loss (%)^4^** | |  |  |  |  |
| < 2.5 | 1.0 (Reference) | 1.0 (Reference) | 1.0 (Reference) | 1.0 (Reference) | 1.0 (Reference) |
| 2.5-6 | 0.85 (0.36-2.03) | 1.46 (0.34-6.21) | 0.72 (0.23-2.22) | 0.51 (0.14-1.82) | 2.03 (0.32-12.90) |
| 6-11 | 0.97 (0.45-2.08) | 0.68 (0.15-3.01) | 0.41 (0.14-1.22) | 0.42 (0.13-1.37) | 0.25 (0.02-3.58) |
| 11-15 | 0.74 (0.35-1.56) | 0.29 (0.06-1.48) | 0.71 (0.27-1.91) | 0.38 (0.12-1.21) | -**^6^** |
| ≥ 15 | 0.80 (0.41-1.57) | 0.67 (0.19-2.34) | 0.53 (0.21-1.29) | 0.39 (0.14-1.05) | 0.72 (0.13-4.00) |
| The category of weight loss and BMI are the same as in the BMI adjusted weight loss grading system.  1 Adjusted for age at surgery, sex, pathological tumor stage, neoadjuvant therapy, Charlson comorbidity index, and tumor histology.  2 Further adjust for preoperative BMI.  3 Further adjust for preoperative weight loss.  4 Further adjust for postoperative BMI and Clavien-Dindo classification.  5 Further adjust for postoperative weight loss and Clavien-Dindo classification.  6 No estimate due to empty cell. | | | | | |
